# Evaluation of spatial dependence of point spread function-based PET reconstruction using a traceable point-like ^22^Na source

**DOI:** 10.1186/s40658-016-0162-3

**Published:** 2016-10-26

**Authors:** Taisuke Murata, Kenta Miwa, Noriaki Miyaji, Kei Wagatsuma, Tomoyuki Hasegawa, Keiichi Oda, Takuro Umeda, Takashi Iimori, Yoshitada Masuda, Takashi Terauchi, Mitsuru Koizumi

**Affiliations:** 1 Department of Radiology, Chiba University Hospital, 1-8-1 Inohana, Chuo-ku, Chiba-shi, Chiba 260-8677 Japan; 2 Department of Radiological Sciences, School of Health Sciences, International University of Health and Welfare, 2600-1 Kitakanemaru, Ohtawara, Tochigi 324-8501 Japan; 3 Department of Nuclear Medicine, Cancer Institute Hospital of Japanese Foundation for Cancer Research, 3-8-31 Ariake, Koto-ku, Tokyo, 135-8550 Japan; 4 Research Team for Neuroimaging, Tokyo Metropolitan Institute of Gerontology, 35-2 Sakae-cho, Itabashi-ku, Tokyo, 173-0015 Japan; 5 School of Allied Health Sciences, Kitasato University, 1-15-1 Kitasato, Minami-ku, Sagamihara, Kanagawa 252-0373 Japan; 6 Department of Neurological Technology, Faculty of Health Sciences, Hokkaido University of Science, 7-Jo 15-4-1 Maeda, Teine, Sapporo, Hokkaido 006-8585 Japan

**Keywords:** Quantitative imaging, PET/CT, Point spread function, Resolution modeling, Spatial dependence

## Abstract

**Background:**

The point spread function (PSF) of positron emission tomography (PET) depends on the position across the field of view (FOV). Reconstruction based on PSF improves spatial resolution and quantitative accuracy. The present study aimed to quantify the effects of PSF correction as a function of the position of a traceable point-like ^22^Na source over the FOV on two PET scanners with a different detector design.

**Methods:**

We used Discovery 600 and Discovery 710 (GE Healthcare) PET scanners and traceable point-like ^22^Na sources (<1 MBq) with a spherical absorber design that assures uniform angular distribution of the emitted annihilation photons. The source was moved in three directions at intervals of 1 cm from the center towards the peripheral FOV using a three-dimensional (3D)-positioning robot, and data were acquired over a period of 2 min per point. The PET data were reconstructed by filtered back projection (FBP), the ordered subset expectation maximization (OSEM), OSEM + PSF, and OSEM + PSF + time-of-flight (TOF). Full width at half maximum (FWHM) was determined according to the NEMA method, and total counts in regions of interest (ROI) for each reconstruction were quantified.

**Results:**

The radial FWHM of FBP and OSEM increased towards the peripheral FOV, whereas PSF-based reconstruction recovered the FWHM at all points in the FOV of both scanners. The radial FWHM for PSF was 30–50 % lower than that of OSEM at the center of the FOV. The accuracy of PSF correction was independent of detector design. Quantitative values were stable across the FOV in all reconstruction methods. The effect of TOF on spatial resolution and quantitation accuracy was less noticeable.

**Conclusions:**

The traceable ^22^Na point-like source allowed the evaluation of spatial resolution and quantitative accuracy across the FOV using different reconstruction methods and scanners. PSF-based reconstruction reduces dependence of the spatial resolution on the position. The quantitative accuracy over the entire FOV of the PET system is good, regardless of the reconstruction methods, although it depends slightly on the position.

## Background

Positron emission tomography/computed tomography (PET/CT) using ^18^F-fluoro-2-deoxy-d-glucose (FDG) has become increasingly important for planning and evaluating the outcomes of treatment [1, 2] and this modality requires accurate quantitation of FDG uptake and distribution related to spatial resolution. However, a limitation associated with the PET/CT system is the degradation of spatial resolution that is attributed to the positron range effect, photon non-collinearity, and detector-related effects including the width of scintillator crystals, inter-crystal scattering, and inter-crystal penetration [3–6]. Detector-response blurring at locations far from the center of the field of view (FOV) is problematic [6, 7], particularly in the radial and axial dimensions.

Alessio et al. characterized the detector response using Monte Carlo simulations [7] and empirical measurements [8]. The detector response in an empirical measurement that separated radial and axial components was modeled as a point spread function (PSF), and PSF information was incorporated into a three-dimensional (3D) iterative reconstruction [4, 6]. A reconstruction algorithm using PSF correction has been reported to improve the spatial resolution in the scanner FOV. Such algorithms are provided from the vendors under different names (SharpIR from GE, TrueX HD·PET from Siemens and ×Sharp from Philips). However, PSF correction changes quantitative accuracy due to the Gibbs ringing overshoot at the edges [4, 9]. Furthermore, although few studies have evaluated the effects of PSF correction using the National Electrical Manufacturers Association (NEMA) body phantom located at the center of the FOV [10, 11], the relationship between FDG uptake at any location throughout the FOV and the accuracy of PSF correction remains unclear. The spatial resolution of the scanner can be measured by placing a point source within the scanner and acquiring scan data at varying locations in both the radial and axial dimension [6, 8].

A point-like ^22^Na source (<1 MBq) that is appropriately related to the national standard in Japan was developed for PET calibration [12, 13]. The radioactivity of this source was calibrated with a Germanium-semiconductor spectrometer at an accredited calibration center (Japanese Radioisotope Association, Tokyo, Japan); the level of uncertainty in the measurement was <1.5 % (*k* = 2). The long half-life of 2.6 years and low maximum positron energy of 0.546 MeV render traceable point-like ^22^Na sources favorable for direct comparisons of spatial resolution and quantitation accuracy among PET scanners [13].

The present study aimed to quantify the effects of PSF correction as a function of the position of a traceable point-like ^22^Na source over the FOV of two types of PET scanners.

## Methods

### Traceable point-like ^22^Na source

The traceable point-like ^22^Na source is described in detail elsewhere [12]. Briefly, the source consisted of a spherical aluminum capsule (outer diameter, 3.0 mm) and a spherical ion exchange resin bead (diameter 0.5 mm) with a surface that absorbs ^22^Na. The influence of absorbed attenuation and scatter was negligible [12, 13]. The amount of source radioactivity was 0.3 MBq at the time of the comparison of PET scanners.

### PET scanner

We used Discovery PET/CT 600 and Discovery PET/CT 710 scanners (GE Healthcare, Milwaukee, WI, USA). Table 1 shows the features of two PET devices compared herein. The Discovery 600 comprises 24 rings of 512 bismuth germinate (BGO) crystals (4.7 × 6.3 × 30 mm), covering transaxial and axial FOV of 700 and 157 mm, respectively. The spatial resolution at 1 cm from the center of the FOV was 4.9 mm at full width at half maximum (FWHM) according to NEMA NU2-2007 [14]. The Discovery 710 comprises 24 rings of 576 lutetium-yttrium oxyorthosilicate (LYSO) crystals (4.2 × 6.3 × 25 mm), covering transaxial and axial FOV of 700 and 157 mm, respectively. The spatial resolution at 1 cm from the center of the FOV was 4.7 mm at FWHM according to NEMA [15].

**Table 1:** Comparison of device features

	Discovery 600	Discovery 710
Transaxial FOV (mm)	700	700
Axial FOV (mm)	157	157
No. of ring	24	24
No. of individual crystals	12,288	13,824
No. of crystals/ring	512	576
No. of image planes	47	47
Crystal size (mm)	4.7 × 6.3 × 30	4.2 × 6.3 × 25
Crystal array per block	8 × 6	9 × 6
Scintillator material	BGO	LYSO
Coincidence window (nsec)	9.5	4.9

### 3D-positioning robot and data acquisition

**Fig. 1:**
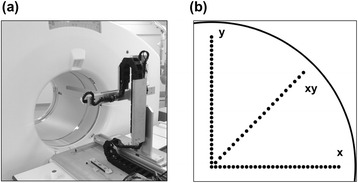
Point-like ^22^Na source setup for PET acquisition. Source is attached to the tip of a robot (**a**) and moved across three directions (**b**)

The point-like ^22^Na source was moved throughout the FOV using a 3D-positioning robot (IAI Co. Ltd., Shizuoka, Japan) that allowed motions with an accuracy of ±0.02 mm (Fig. 1a). The robot was controlled by coded software running on a personal computer. The orientation accuracy of robot positioning was verified before each acquisition in three dimensions [6]. Data acquisition in each direction started with the source positioned at the center of the FOV. The source was then moved to the peripheral 30 cm of the transaxial FOV at 1 cm intervals in the directions of the *x*-, *y*-, and *xy*-axes (Fig. 1b). The *xy*-axis was moved in an oblique 45° direction, for example (*x*,*y* = 0, 0), (*x*,*y* = 1, 1), and (*x*,*y* = 2, 2), namely, at √2-cm intervals. PET data were acquired for 2 min at each point in 3D list mode.

### Image reconstruction

We reconstructed PET data using the following algorithms: filtered back projection (FBP), baseline ordered subset expectation maximization (OSEM), OSEM + PSF, and OSEM + PSF + time-of-flight (TOF; Discovery 710 only). The PSF modeling used in this study was the SharpIR algorithm conceived by Alessio et al. [7, 8]. Thirty-one data series for the *x*- and *y*-axes and 22 data series for the *xy*-axis divided into each measured point were reconstructed. The iterative reconstruction parameters of the Discovery 600 were 2 iterations and 16 subsets for OSEM, and 3 iterations and 16 subsets for OSEM + PSF. The iterative reconstruction parameters of the Discovery 710 were 2 iterations and 18 subsets for OSEM, and 3 iterations and 18 subsets for OSEM + PSF and OSEM + PSF + TOF. The image matrix was 256 × 256 (pixel size, 2.73 mm) and slice thickness was 3.27 mm. A smoothing filter was not used for post-processing. Attenuation and scatter were not corrected. The normalization scan was performed using a uniform cylindrical ^18^F-filled phantom located at the center of the FOV.

### FWHM analysis

The spatial resolution in the scanner FOV was determined from the FWHM of the PSF obtained from the activity distribution from the point-like ^22^Na source. The FWHM in three directions for each measured point was calculated according to the NEMA requirements (NEMA-NU2-2012) [16]. The maximum value of the PSF was determined using a parabolic fit of the peak point and its two nearest-neighbor points. The position at half of the profile maximum value was determined by linear interpolation between adjacent pixels. The pixel size was set to 1.56 mm by reducing the reconstructed FOV width when evaluating the FWHM.

### Quantitative analysis

Data were analyzed using OsiriX software (Pixmeo, Bernex, Switzerland). Circular regions of interest (ROI) were defined around the source position to obtain the total ROI value. We placed ROI on 21 image planes around the central image plane within a range of ±10 image planes to cover the entire smeared radioactivity in the axial direction. The sum of the ROI values derived from 21 image planes was defined as ROI_total_. Scattered 1.275-MeV γ rays along with β+ decay of ^22^Na might increase ROI values when the radius of the ROI is larger. In the present study, the ROI radius was set to 16 mm so that the uniformity in the field of view was evaluated using the traceable point-like source in a way that is independent of the partial volume effect, on the basis of a previous study [13]. The recovery coefficient (RC), a function of radial distance from the center of the FOV, served as a quantitative index [14], as follows:

$$ \mathrm{R}\mathrm{C} = {\mathrm{ROI}}_{\mathrm{total}} \times {\nu}_{\mathrm{pixel}}/\ \left({R}_{\mathrm{source}}/\varepsilon \right) $$

where *ν*_pixel_ is the volume of pixel in cc, *R*_source_ is the emission rate of 0.511-MeV γ rays from the ^22^Na source in s^−1^, and *ε* is the β^+^ branching ratio of ^22^Na.

## Results

The images of the point source for FBP and OSEM were broad and faint when located far from the center of the FOV, whereas hot spots for PSF and PSF + TOF became sharp and dense throughout the FOV. The trends of results from the Discovery 600 and 710 scanners were almost identical.

**Fig. 2:**
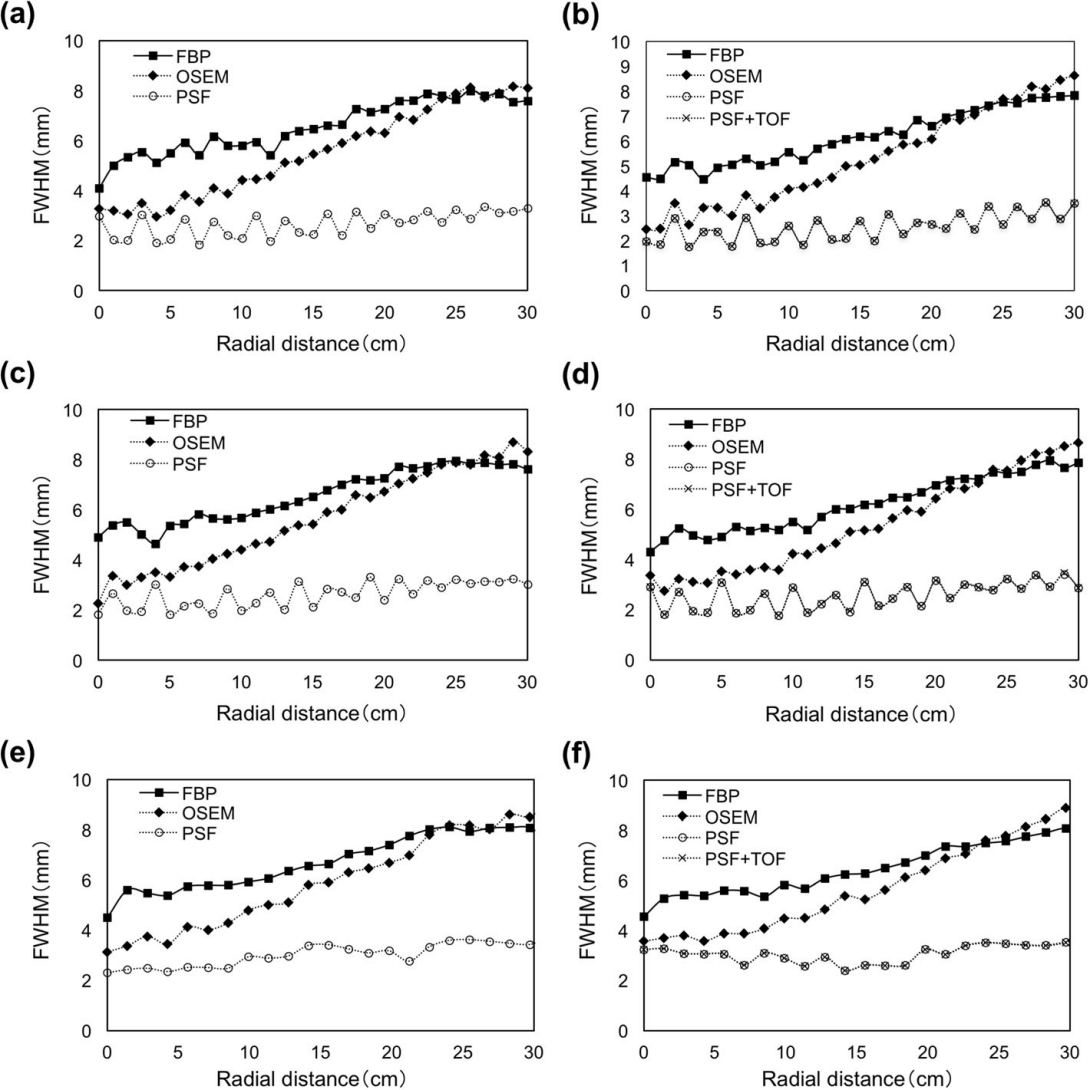
Radial FWHM at different radial locations for *x*- (**a**, **b**), *y*- (**c**, **d**), and *xy*- (**e**, **f**) axes using Discovery 600 (**a**, **c**, **e**) and Discovery 710 (**b**, **d**, **f**)

**Fig. 3:**
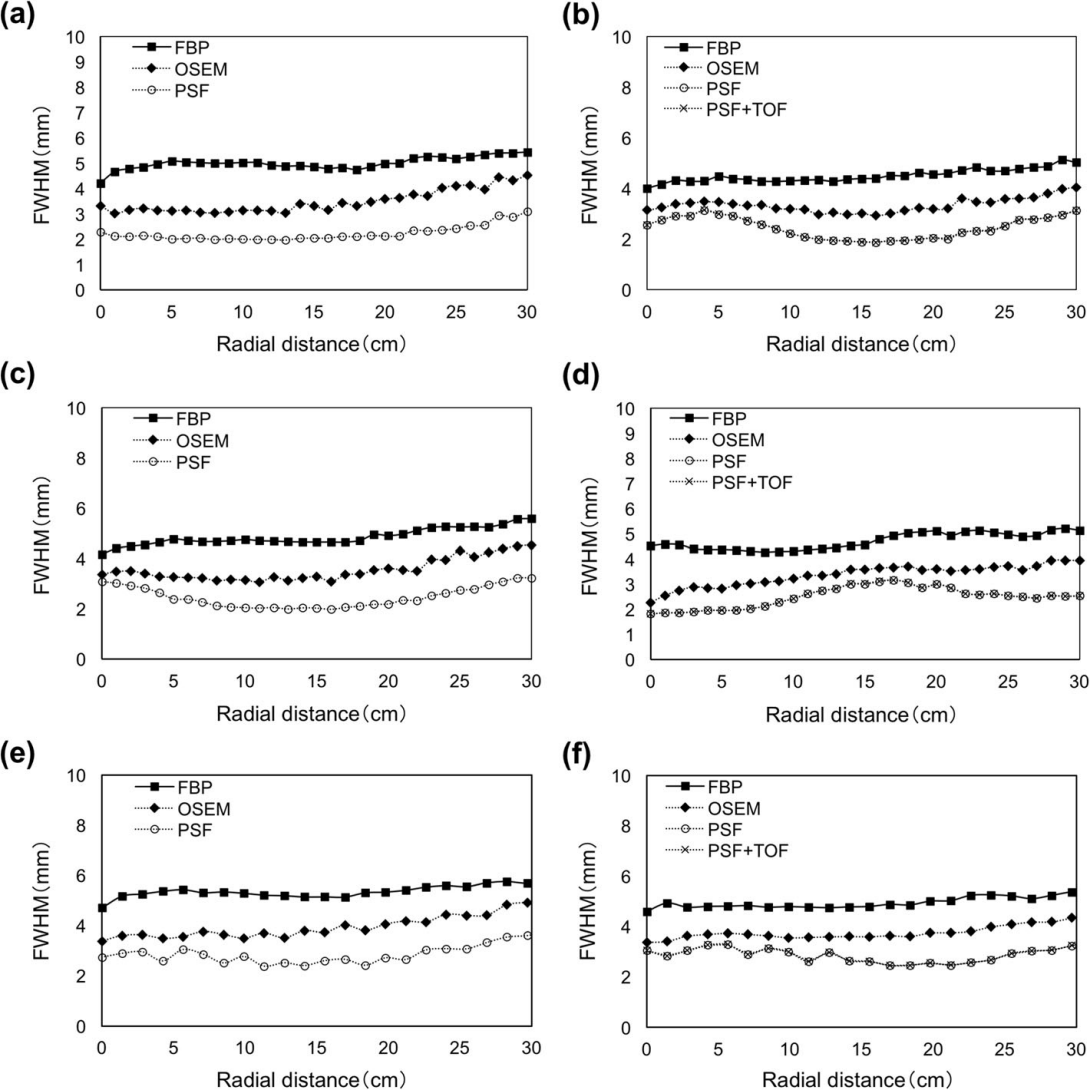
Tangential FWHM at various moving radial locations for *x*- (**a**, **b**), *y*- (**c**, **d**), and *xy*- (**e**, **f**) axes using Discovery 600 (**a**, **c**, **e**) and Discovery 710 (**b**, **d**, **f**)

Figure 2 shows the radial FWHM as a function of radial distance from the center of the FOV in three directions. The radial FWHM for FBP and OSEM increased towards the periphery of the FOV, whereas PSF and PSF + TOF recovered and spatial resolution was more uniform over the entire FOV. The FWHM data were similar among the measured directions and scanners. The results of PSF and PSF + TOF were identical using the Discovery 710. The radial FWHM for PSF and PSF + TOF increased and decreased in a cyclic fashion across the FOV. Figure 3 shows the tangential FWHM. Regardless of reconstruction methods, the tangential FWHM were stable at all positions. The tangential FWHM of PSF and PSF + TOF were smaller than those of FBP and OSEM.

**Fig. 4:**
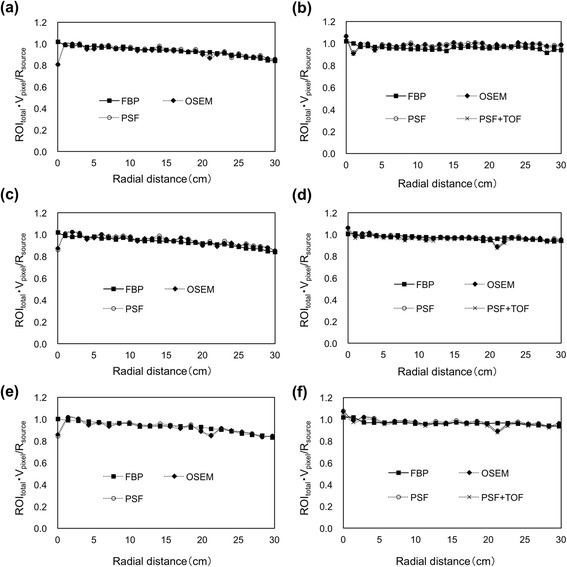
Recovery coefficient at various radial locations for *x*- (**a**, **b**), *y*- (**c**, **d**), and *xy*- (**e**, **f**) axes using Discovery 600 (**a**, **c**, **e**) and Discovery 710 (**b**, **d**, **f**)

Figure 4 shows that the RC as a function of radial distance from the center of the FOV in three directions, remained stable across the FOV in all reconstruction methods, in particular for the Discovery 600, and fluctuated only at the center of the FOV in both scanners.

## Discussion

We evaluated the spatial dependence of PSF-based PET reconstruction using a traceable ^22^Na point-like source on two PET scanners with different detector designs. The results showed that PSF correction improves spatial resolution and does not affect quantitative values over the entire FOV.

The methodology used to measure the response of the PET system that determines the spatial dependence of the PSF inside the FOV is important. Several factors must be considered to obtain an accurate response from the PET system, such as the source dimensions, type of isotope, media surrounding the point source (air, water, or warm radioactive background), and the number and position of measurements to account for all possible responses of the system [17]. In particular, attenuation and scattering owing to the source (phantom) and its correction produce uncertainty in performance evaluations. We adopted a ^22^Na point-like source that is traceable to a national standard, and a 3D-positioning robot. Although the ^22^Na point-like source has been proposed for PET calibration [12, 13], it is also useful for evaluating the intrinsic characteristics of different PET scanners in terms of responses to the source in a manner that is independent of uncertainty caused by attenuation and scatter.

The radial FWHM for FBP and OSEM increased to approximately 4 and 5 mm, respectively, for the scanner FOV at 30 cm, whereas tangential FWHM was more or less independent of the radial offset (Figs. 2 and 3). Both PSF and PSF + TOF achieved a radial FWHM in the peripheral FOV that was equivalent to the center of FOV, and the FWHM was uniform (approximately 2 mm) across the FOV in both scanners. Although this FWHM was 30–50 % lower than that of OSEM at the center of FOV, evaluation with the point source in air might have caused PSF over-correction of resolution because of unacceptable noise properties, which is a clinically unrealistic scenario. Alessio et al. found more modest resolution gains with PSF correction when sources were placed in a warm background [8]. On the other hand, the FWHM were similar between the Discovery 600 and 710 scanner using PSF correction. Wiant et al. examined the spatial dependence of PSF-based reconstruction in 2D mode (with septa) using a Discovery ST PET/CT scanner (GE Healthcare), thus validating the accuracy of the PSF correction across the FOV [6]. The accuracy of PSF correction was independent of detector design, possibly because the detector-response function was determined by optimizing each type of scanner.

The radial FWHMs for PSF and PSF + TOF slightly increased and decreased in a cyclic fashion across the FOV (Fig. 2). This could be explained by the location of the point source within a pixel. The maximum pixel value was underestimated due to the position of the source relative to the grid of pixel separation [18]. The point spread function of hotspots became deformed (sharp and dense) by PSF correction, and thus the location of a point source in a pixel is thought to considerably influence the FWHM. This problem can be addressed to decrease the FOV or increase the matrix size.

Time-of-flight could reduce axial blurring to recognize the correct axial plane of origin for each line-of-response (LOR) [19]. However, TOF did not affect the spatial resolution of the entire transaxial FOV (Figs. 2 and 3). The FWHM of spatial uncertainty using TOF was around 7–8 cm since the time resolution of LYSO was 500 psec; thus, TOF does not directly improve spatial resolution [20].

Quantitative performance among different reconstructions across FOV has not previously been evaluated. The RC was stable across the FOV in all reconstruction methods using both scanners. We calculated the total ROI values covering the traceable ^22^Na point-like source to create a quantitative index. Although the degree of smearing of radioactivity differed depending on the reconstruction methods, ROI_total_ remained constant across all reconstruction methods. We found that PSF correction does not impact the total quantified value regardless of changes in peak height and the shape of the PSF. The integral of the modeled PSF normally equals unity and therefore it has no effect on the total reconstructed activity, only on how exactly that activity is distributed in the PET image. This is confirmed by the results that the curves for OSEM, OSEM with PSF, and OSEM with TOF and PSF are identical. The RC fluctuated only at the center of FOV in both scanners. The uncertainty of measurement at the center of the FOV in PET is due to a geometric factor, which principally results in avoiding taking measurements at the center of the FOV during quantitative analyses [13]. The RC of PSF and PSF + TOF were identical. The point-like ^22^Na source was small and independent of absorber attenuation and scatter, and thus TOF gain was not obtained [20].

The results shown in Fig. 4 revealed that the quantification is indeed position dependent, with excellent performance near the center of the FOV, and a slight underestimation near the edge of the FOV. The detector efficiency variations and geometric factors such as the solid angle subtended are typically corrected by the normalization scan for the uniformity across the FOV [21]. Nevertheless, the RC decreased slightly towards the periphery of the FOV in all reconstruction methods. We consider that this might be due to the uncertainty of a standard normalization method using cylindrical water phantoms and the uncertainty of attenuation and scatter corrections.

The present study has several limitations. First, several PSF modeling with different principles are provided by vendors. Differences in PSF modeling might have influenced the results. Further study with other PSF modeling algorithms is required to assess the relationship between the location of FDG uptake inside the FOV and the accuracy of PSF correction. Second, we evaluated spatial resolution and quantitative accuracy using only one point source in air. A point source in air is a very artificial object, very different from the tracer distributions in human bodies which are seen in clinical routine. Further study should evaluate the influence of attenuation and scattering using a hot point source surrounded by activity and a phantom with hot spheres of different sizes in a warm background in a more realistic imaging scenario to verify the advantages and disadvantages of PSF and TOF correction in the FOV periphery [22].

## Conclusions

A traceable ^22^Na point-like source allowed evaluations of spatial resolution and quantitative accuracy among different reconstructions across the FOV. We found that PSF-based reconstruction reduces dependence of the spatial resolution on the position. The quantitative accuracy over the entire FOV of the PET system is good, regardless of the reconstruction methods, although it depends slightly on the position. PSF correction might benefit the acquisition of PET images from obese patients that occupy most of the FOV and of small or distal lesions such as nodal metastases [23].
